# Immunocompetent hamsters as a model for orthobunyavirus-induced neuroinvasion and neuropathology

**DOI:** 10.1371/journal.pntd.0011355

**Published:** 2023-05-26

**Authors:** Allison Groseth, Don Gardner, Kimberly Meade-White, Susanne Amler, Hideki Ebihara

**Affiliations:** 1 Laboratory of Virology, Division of Intramural Research, National Institute of Allergy and Infectious Diseases, National Institutes of Health, Hamilton, Montana, United States of America; 2 Laboratory for Arenavirus Biology, Institute of Molecular Biology and Cell Biology, Friedrich-Loeffler-Institut—Federal Research Institute for Animal Health, Greifswald—Insel Riems, Germany; 3 Rocky Mountain Veterinary Branch, Division of Intramural Research, National Institute of Allergy and Infectious Diseases, National Institutes of Health, Hamilton, Montana, United States of America; 4 Friedrich-Loeffler-Institut—Federal Research Institute for Animal Health, Greifswald—Insel Riems, Germany; 5 Department of Virology 1, National Institute of Infectious Diseases, Tokyo, Japan; NIAID Integrated Research Facility, UNITED STATES

## Abstract

**Background:**

Bunyavirus infections, including those caused by Bunyamwera serogroup orthobunyaviruses, represent a significant and yet likely still vastly underappreciated cause of mild to moderate human febrile infections. In severe cases, these infections can also cause neurological disease, particularly meningitis and encephalitis, and infection can even be fatal. However, with a few exceptions, information regarding the mechanisms underlying the neuroinvasion and neuropathogenesis of such infections is limited. This is due in part to a lack of animal models to facilitate such studies.

**Methodology/Principal findings:**

In an effort to develop an immunocompetent model of infection with Bunyamwera serogroup orthobunyaviruses, we infected 4-6-week-old female hamsters via either the intraperitoneal or subcutaneous route with 10^6^ pfu/animal of Bunyamwera virus (BUNV), Batai virus or Ngari virus. Only BUNV infection resulted in clinical disease, which was characterized by weight loss, lethargy and neurological signs (i.e. tremor of the head or limbs, loss of righting reflex, “waltzing”). While symptoms were of similar severity for both routes, they occurred more frequently following subcutaneous inoculation. Consistent with these clinical signs, both antigen staining and histopathological abnormalities were found extensively throughout the brain.

**Conclusions/Significance:**

The reported hamster model of BUNV infection provides a new tool for studying orthobunyavirus infection, and particularly neuroinvasion and the development of neuropathology. This model is particularly significant because it makes use of immunologically competent animals and relies on a subcutaneous inoculation route that more closely mimics the natural infection route for arboviruses, thereby providing a more authentic cellular and immunological context at the initial site of infection.

## Author summary

Bunyaviruses cause a significant number of human infections, and while most are relatively benign, in severe cases encephalitis and/or meningitis can develop. Little is known about the biological processes that allow bunyaviruses to enter the brain, and why this results in neuropathology in some cases. Available models are frequently based on direct intracranial infection in animals with impaired or underdeveloped immune systems. Here we have infected subadult hamsters via either the intraperitoneal or the subcutaneous route with one of three different bunyaviruses: Bunyamwera virus (BUNV), Batai virus or Ngari virus. Only BUNV infection resulted in clinical disease, with infected animals exhibiting weight loss, lethargy and neurological signs of disease. Consistent with these observations pathological abnormalities and viral antigen were found extensively throughout the brain. The establishment of this new model is significant because it makes use of immunologically competent animals and relies on an inoculation route that more closely mimics the natural route of infection through insect bites. As such, it provides an important basis for studying neuroinvasion and the development of neuropathology during bunyavirus infection as well as for the testing of antiviral approaches targeting these viruses.

## Introduction

The recently created family *Peribunyaviridae* encompasses a large and highly diverse group of single-stranded negative-sense RNA viruses whose genomes are divided into 3 segments: the S-segment, M-segment and L-segment. To date only members of the genus *Orthobunyavirus*, which currently contains over 100 recognized species [1], have been shown to infect humans or livestock species. While members of the California serogroup, which are an endemic cause of encephalitis in North America, are generally considered the most medically significant of these viruses with respect to human disease, members of the Bunyamwera serogroup are also associated with human and animal disease [2]. Bunyamwera serogroup viruses can be found throughout much of the world, although individual viruses have more restricted geographical distributions, where they are transmitted mainly by mosquitoes and, in a few cases, also by biting midges [3]. Unfortunately, our understanding of the prevalence of and disease burden posed by Bunyamwera serogroup viruses is currently limited, primarily due to a lack of systematic data collection.

The prototype for this group, Bunyamwera virus (BUNV), occurs throughout sub-Saharan Africa where the human antibody prevalence in some regions has been reported to be >50% [4]. Based on the small number of cases for which clinical data is available, BUNV infection in otherwise healthy adults appears to result mainly in a mild self-limiting infection accompanied by fever, headache, arthralgia and rash [4,5]. However, a substantial proportion of these cases also report central nervous system involvement in the form of visual disturbances and vertigo, which represents a so far underappreciated aspect of the disease [4]. Further, in immunocompromised patients, BUNV infection can result in severe encephalitis and/or meningitis [6]. In contrast to this, infection with a related virus, Batai virus (BATV), has only been reported to cause a non-descript febrile illness, in some cases with a respiratory and/or gastrointestinal component [7]. To date no neurological component to the disease has been described in humans, although encephalitis was reported recently in an infected harbor seal [8]. BATV is found throughout Europe and Asia with variable rates of seroprevalence in humans (from <1–32%), that can also remain low despite high rates of infection in livestock in these same regions [2,9–11]. Surprisingly, Bunyamwera serogroup viruses have also shown the ability to dramatically increase their pathogenicity through reassortment. This was observed for a naturally occurring reassortant of BUNV and BATV (S_BUNV_/M_BATV_/L_BUNV_) [12,13] known as Ngari virus (NRIV), which is found in Central Africa where it causes fatal hemorrhagic fever (HF) [12,14–16].

Despite an increasing appreciation of their connection to sometimes-severe disease outcomes, at present little is known about either the virulence determinants or the pathophysiological processes that underlie the development of disease during infection with Bunyamwera serogroup viruses. In particular, a major challenge for such studies, is that those models that have so far been reported to result in severe disease/death are based on direct intracranial inoculation, usually using immunologically immature or immunodeficient animals, which bypasses the need for neuroinvasion from peripheral infection sites [17–22]. However, past successes using hamsters to model the infections caused by other encephalitic and HF-causing viruses (e.g. [23–28]), suggested this might also be a viable approach for modelling bunyavirus infections associated with these severe disease phenotypes. Indeed, anecdotal reports have even suggested that peripheral infection of hamsters with BUNV may result in lethal disease, specifically a fatal encephalitis, but the details of such a model and its clinical outcome have never been reported [19].

We, therefore, sought to establish whether infection of hamsters can indeed serve as a model for the development of severe disease manifestations associated with orthobunyavirus infection (i.e. neuropathology and/or hemorrhagic fever). To do so we infected groups of Syrian golden hamsters with BUNV, BATV or NRIV and monitored the development of clinical signs and characterized the accompanying pathology in these animals. Based on the results we could indeed confirm that infection with BUNV results in a partially lethal model of BUNV-induced neurological disease following peripheral inoculation.

## Methods

### Ethics statement

Animals for immunization were housed in the Rocky Mountain Laboratories (RML) BSL-2 animal facility and research was conducted under protocol #2011–68.11. Animals for infection studies were housed in the RML BSL-4 high biocontainment laboratory and research was conducted under protocol #2012-053-E. Both protocols were approved by the National Institute of Allergy and Infectious Diseases/RML Institutional Animal Care and Use Committee in compliance with their guidelines. The facility where this research was conducted is fully accredited by the Association for the Assessment and Accreditation of Laboratory Animal Care International and has an approved Office of Laboratory Animal Welfare Assurance (#A4149-01). All procedures were conducted by trained personnel and all invasive clinical procedures were performed while animals were anesthetized. Early endpoint criteria, as specified by the Institutional Animal Care and Use Committee approved scoring parameters, were used to determine when animals should be humanely euthanized.

### Cell lines and viruses

VeroE6 (African green monkey kidney) cells were maintained in Dulbecco’s modified Eagle’s medium (DMEM, Life Technologies) supplemented with 10% fetal bovine serum (FBS, Life Technologies), 2 mM L-glutamine (Q; Life Technologies), 100 U/mL penicillin and 100 μg/mL streptomycin (PS; Life Technologies) and grown at 37°C with 5% CO_2_.

BUNV (strain 6547–8) and BATV (strain UgMP 6830) were kindly provided by Bob Tesh at the World Reference Center for Emerging Viruses and Arboviruses (WRCEVA), while NRIV (strain 9800535) was kindly provided by Pierre Rollin and Stuart Nichol at the Centers for Disease Control and Prevention (Table 1). The BUNV (strain 6547–8) isolate used in this study had been previously passaged 47 times in suckling mouse brain and once in Vero cells and was then passaged twice more on VeroE6 cells for preparation of working stocks. The BATV (strain UgMP 6830) isolate used had been previously passaged 4 times in suckling mouse brain and was passaged three times on VeroE6 cells for preparation of working stocks. The NRIV strain used was initially isolated by growth on Vero cells [14] and was subjected to 2 further passages on VeroE6 cells for preparation of our working stocks. The identity of these virus stocks was confirmed by complete genome sequencing before use. In the case of BATV and NRIV, these sequences have been previously published [29,30], while for BUNV the resulting sequences were found to be nearly identical to the GenBank reference sequence for BUNV (strain 6547–8) provided in Table 1 across all three segments (S: 99.8%, M: 99.5%, L: 99.9% nucleotide identity).

**Table 1:** Challenge virus strains.

Virus Species	Strain	Strain Information			
		Isolation Date	Location	Source	GenBank Accession Number
Bunyamwera virus	6547–8	1943	Uganda	*Aedes sp*.	S: NC_001927 M: NC_001926 L: NC_001925
Batai virus	UgMP 6830	1968	Uganda	*Aedes abnormalis*	S: JX846601 M: JX846602 L: JX846603
Ngari virus	9800535	1998	Kenya	Human (Hemorrhagic Fever)	S: JX857328 M: JX857329 L: JX857330

All work with NRIV, as well as infection experiments with all three viruses, were performed in the BSL-4 high biocontainment laboratory at Rocky Mountain Laboratories (Division of Intramural Research, National Institute of Allergy and Infectious Diseases, National Institutes of Health).

### Infection of hamsters

Four- to six-week-old female Syrian golden hamsters (*Mesocricetus auratus*) were purchased from a commercial supplier (Harlan Laboratories, now Envigo) and allowed to acclimate to the high biocontainment laboratory for one week prior to the beginning of the experiment. Animals were ear tagged for identification and housed with 3 animals per cage (or 2 for control groups) in disposable IVC cages as part of the Innovive IVC rat caging system with aspen chip bedding (P.J. Murphy’s Sani-Chips). They were fed with 2016 Teklad rodent chow (Envigo) provided *ad libitum* via the overhead wire bar food hopper. Groups of 6 individuals were infected i.p. with 1x10^6^ pfu of BUNV (strain 6547–8), Batai virus (strain UgMP6830) or NRIV (strain 9800535) in a total volume of 400μl DMEM delivered as injections at two adjacent sites in the lower right abdominal quadrant. Alternatively, groups were infected s.c. with 1x10^6^ pfu of virus in a 200ul volume as a single injection at the back of the neck. Control animals (total n = 4) received DMEM in the same volumes either s.c. (n = 2) or i.p. (n = 2) as described above. All groups had comparable average starting weights (range: 72.6–80.7 g). Given that this was an exploratory study for which information regarding outcome was unavailable, group sizes were selected based on the Resource Equation Method [31].

Hamsters were monitored daily for weight loss and signs of disease. Animals were euthanized when they met the humane end-point criteria defined by the animal use protocol (i.e. weight loss >20%, neurological signs or hemorrhagic signs of disease). Organ samples (i.e. liver, spleen and brain) were collected from selected animals at the time of euthanasia and processed for histopathological analysis as described below. Blood was also collected via cardiac puncture at the time of euthanasia and stored at -80°C until processed for plaque assay, also as described below. All surviving animals were euthanized at day 21 post-infection.

### Recombinant BUNV N expression and antibody generation

To allow expression of recombinant BUNV N without the additional expression of NSs, the pair of adjacent alternate downstream ATGs responsible for NSs translation were abolished by site-directed mutagenesis of pATX-BUNV N (i.e. T106C; T109C). This resulted in silent mutation at the corresponding sites in the N ORF (i.e. HD>HD). The resulting NΔNSs-coding sequence was then subcloned into the pGEX-6P-1 vector (GE Healthcare) for protein expression in *E*. *coli* strain BL-21 (GE Healthcare) following induction using 0.1mM isopropyl β-D-1-thiogalactopyranoside (IPTG) according to the manufacturer’s instructions (GE Healthcare). Purification via affinity chromatography was performed as previously described [32]. Briefly, the bacteria were lysed using chicken egg white lysozyme (Sigma) and sonication, after which purification was achieved using batch binding to glutathione-sepharose 4B resin, also according to the manufacturer’s directions (GE Healthcare).

For polyclonal antibody production, two 10-month-old adult female guinea pigs received injections containing 1ug purified recombinant GST-BUNV N emulsified 1:1 with TitreMax Gold (Sigma) intramuscularly in each caudal thigh in a volume of 100ul. Two additional booster immunizations were performed at 4-week intervals in the same manner but using incomplete Freund’s adjuvant (Sigma). Ten days after the last immunization, blood was collected using vacutainer serum separation tubes (BD), which were incubated for 20 min at 37°C before being centrifuged at 1100 x g for 10 min. The resulting serum fraction was collected, aliquoted and stored at -80°C until use.

### Histopathology and immunohistochemistry

Collected tissues were fixed at 4°C for a minimum of 7 days in 10% neutral buffered formalin before being transferred to fresh formalin for removal from the high biocontainment laboratory. Tissues were then placed in cassettes and processed with a VIP-5 Tissue Tek processor (Sakura Finetek) using a graded series of ethanol, xylene, and ParaPlast Extra. Embedded tissues were sectioned with 5μm thickness and dried overnight at 42°C prior to staining with hematoxylin/eosin. Alternatively, for immunohistochemistry, the tissues were processed using the Discovery XT automated stainer (Ventana Medical Systems) with a DABMap kit (Ventana Medical Systems) using a polyclonal guinea pig anti-BUNV NΔNSs primary antibody (produced as described above; 1:100 dilution), a Biogenex biotinylated anti-guinea pig secondary antibody and hematoxylin counter-staining. Samples were evaluated by a Board-Certified Pathologist for signs of necrosis and/or inflammatory cell infiltration.

### Plaque assay

VeroE6 cells were seeded into 24-well plates the day before titration. Blood samples were thawed and serial dilutions (10^−1^ to 10^−6^) were prepared in DMEM without FBS. Medium was removed from cells and wells were inoculated with 1ml of each virus dilution. After one hour, the inoculum was removed and wells were overlaid with a 1:1 mixture of 2x MEM supplemented with 4% FBS, 2x Q and 2x PS with 1.8% UltraPure Agarose (Life Technologies). Cells were incubated at 37°C for 3 days before the addition of 10% formalin containing 0.1% (w/v) crystal violet. After overnight incubation, the crystal violet solution and agarose overlays were removed and plaques counted.

### Statistical methods

Survival analyses are presented as Kaplan-Meier curves and were analyzed using two-sided log-rank tests. Average weight change was compared using linear mixed models with fixed and random coefficients to account for the structure of longitudinal data when repeated measurements were made on the same subject over time and to account for possible dropout of subjects at different time points. Data analyses have to be considered exploratory and thus no adjustment for multiplicity was performed. Data analyses was performed using the R software [33] and Graphpad PRISM with p-values ≤ 0.05 considered significant.

## Results

### Clinical observations

Infection of subadult (5-7-week-old) female hamsters with BUNV, BATV or NRIV was carried out by either the s.c. or i.p. route using 1x10^6^ pfu of the respective virus. All BATV- and NRIV-infected hamsters survived the entire 21-day course of the experiment during which time the only evidence of illness was a modest delay in weight gain compared to the respective control groups (S1 and S2 Figs), which was statistically significant for the BATV s.c. (p = 0.01), NRIV s.c. (p = 0.005) and NRIV i.p. (p = 0.03) groups.

**Fig 1:**
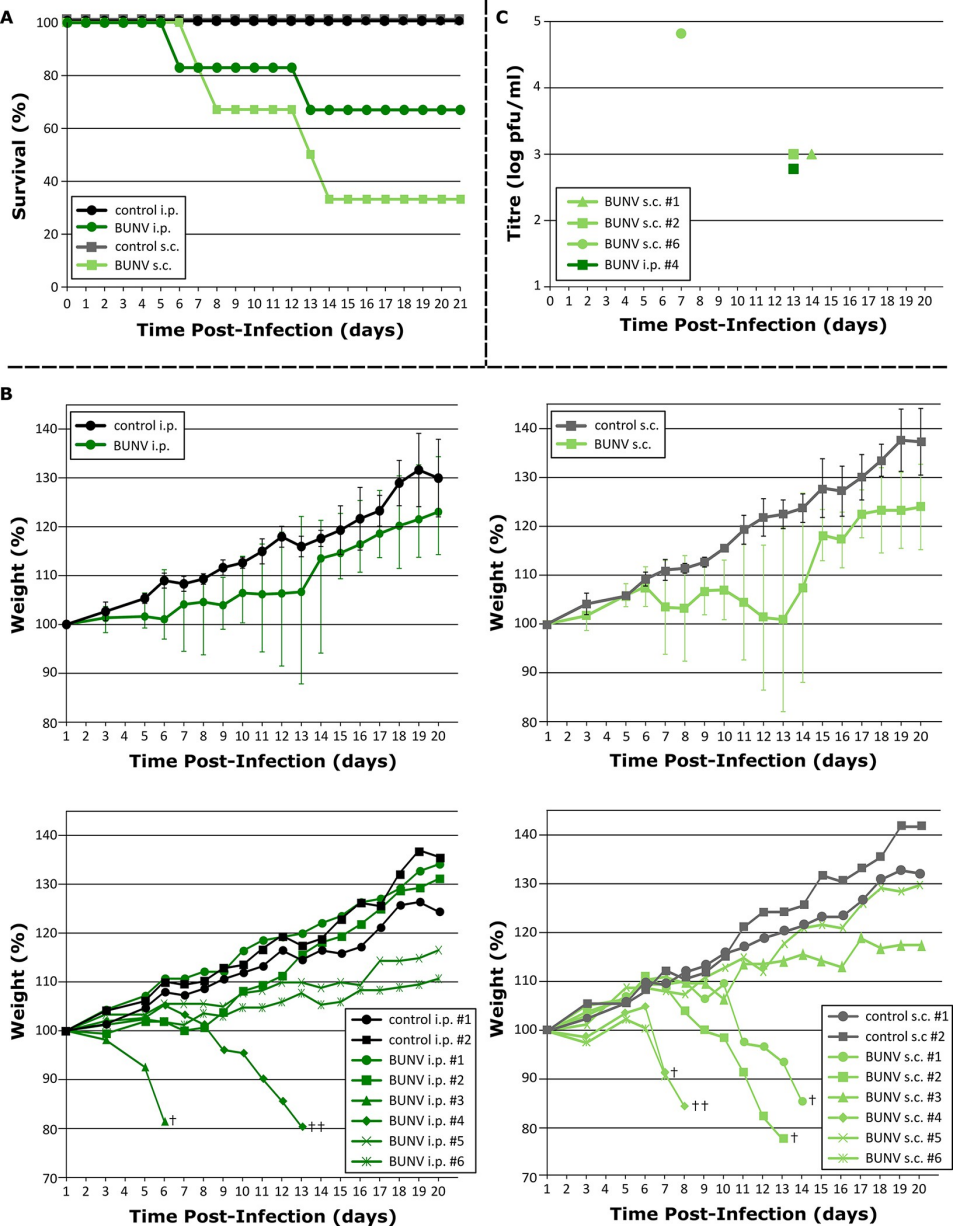
Survival and weight loss in Bunyamwera virus (BUNV)-infected hamsters. Groups of 6 Syrian golden hamsters were inoculated by either the intraperitoneal (i.p.) or subcutaneous (s.c.) route with 10^6^ plaque-forming units of BUNV. **(A) Survival in infected hamsters.** Kaplan-Meier survival curves show the survival of the animals in each group over time. **(B) Weight loss in infected hamsters.** Change in weight over time is shown for both i.p. infected (left panels) and s.c. infected (right panels) animals as both the group mean ± standard deviation (upper panels), and as individual animal weights (lower panels). A single dagger (†) indicates animals from which blood samples were collected for virus titration at the time of euthanasia, while double daggers (††) indicate animals from which samples were collected for histopathology. **(C) Viremia at the time of euthanasia in diseased animals.** Blood samples were collected from the animals indicated in (B) upon reaching the euthanasia criteria and titrated by plaque assay to determine virus load.

In contrast, among BUNV-infected hamsters, 2 of 6 animals in the i.p. group and 4 of 6 animals in the s.c. group fulfilled the pre-determined euthanasia criteria (Fig 1A) based on a combination of severe weight loss and neurological disease signs. Not only did the affected animals fail to gain weight normally, but they showed profound and rapid weight loss (Fig 1B). Differences in weight gain over time were statistically significant for BUNV s.c. infected animals in comparison to route-matched controls (p = 0.028); however, differences were not significant for the BUNV i.p. group due to the high variability in outcome among animals (i.e. 2/6 with profound weight loss, 2/6 with delayed weight gain, and 2 with essentially normal growth). Weight loss in both groups was usually accompanied by lethargy and, in some animals, also hunched posture, lack of muscle tone and/or delayed righting. These findings represented the first indications of overt disease after which affected animals invariably progressed to fulfill the euthanasia criteria (Fig 1A). Euthanasia occurred on days 7, 8, 13 and 14 in the s.c. group and on days 6 and 13 in the i.p. group. At the time of euthanasia, weight loss from 9.1–22.4% was observed (Fig 1B). However, excessive weight loss alone represented the sole euthanasia criteria for only a single animal in this study (Fig 1B, left panel, BUNV i.p. #4), and even in this animal a possible transient torticollis had been observed. All other diseased animals exhibited clear neurological signs of disease. These included tremors of the head (BUNV s.c. #6) or front paws (BUNV s.c. #1), waltzing (counter-clockwise; BUNV s.c. #2, #4, #6) and face pressing (BUNV s.c. #4), and in one animal, an apparent seizure leading to an inability to right coupled with continuous dyscoordinated kicking of one hind leg (BUNV i.p. #3). Interestingly, prior to the onset of other definitive neurological signs, one animal exhibited hyper-excitability when disturbed despite otherwise being markedly lethargic (BUNV s.c. #2). Overall, the clinical observations (including time to death) in diseased BUNV-infected animals were consistent between the two infection routes examined, but the frequency with which animals developed disease was higher in the s.c. group (4/6 animals) compared to the i.p. group (2/6 animals) (Fig 1A and 1B). Due to the small number of control animals included in this initial exploratory study, and the mixed outcomes observed among infected animals, statistically significant differences in survival could not be demonstrated for either the BUNV i.p. or s.c. infected groups even after combining the i.p. and s.c. control animals, although this generated values bordering on significance at the p≤0.05 level (i.e. p = 0.053) for the BUNV s.c. infected animals.

### Gross pathology and virology

A complete necropsy was performed on two of the six diseased BUNV-infected animals at the time of euthanasia (indicated in Fig 1B). No gross pathological changes were observed in the visceral organs of either of the necropsied animals beyond a slight splenomegaly in BUNV s.c. #4. However, this was not sufficiently pronounced as to be clearly pathological and/or associated with the experimental viral infection. Blood was collected via cardiac puncture at the time of euthanasia for analysis by plaque assay from the other 4 BUNV-infected animals showing overt disease. The animals sampled all demonstrated viremia, with titres of between 6x10^2^ and 7x10^4^ pfu/ml at the time of death, with the highest titre observed in the animal that succumbed more rapidly to death (i.e. at 7 dpi vs 13–14 dpi for the other animals tested) (Fig 1C).

### Histopathological observations

**Fig 2:**
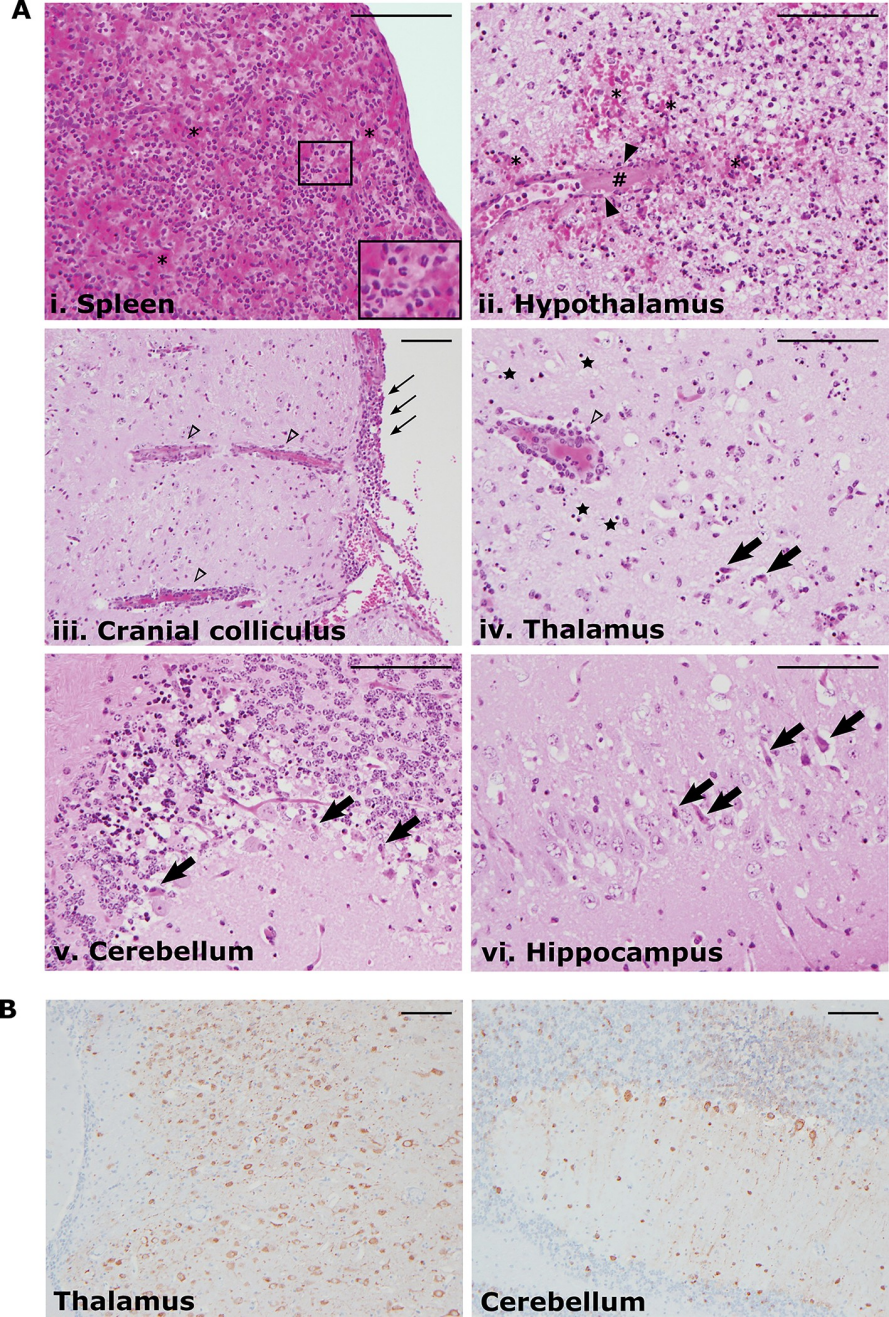
Histopathological changes in tissues from BUNV-infected hamsters. **(A) Hematoxylin/eosin staining.** Selected organs were collected from BUNV-infected animals at the time of euthanasia and fixed in 10% formalin after which they were processed for sectioning and straining with hematoxylin/eosin. (i) Splenic red pulp with an inset showing neutrophil infiltration, (ii) hypothalamus/ventral midbrain, (iii) cranial colliculus, (iv) thalamus, (v) cerebellum and (vi) hippocampus are shown. Major pathological observations are indicated as follows: erythrocyte congestion/hemorrhage (*), thrombus formation (#), vascular necrosis (solid arrow heads), perivascular cuffing (open arrow heads), leptomeningitis (thin arrows), neuronal cell death (thick arrows), glial cell death (black stars). **(B) Immunohistochemical labelling for BUNV antigen.** Selected brain sections from BUNV-infected animals were fixed and sectioned as in (A) before being incubated with a polyclonal guinea pig anti-BUNV N antibody, secondary biotin-labelled anti-guinea pig antibody and 3,3′-Diaminobenzidine (DAB) chromogen. Scale bars indicate 100μm.

Selected visceral tissues (i.e. spleen and liver), as well as whole brains, were collected from the necropsied BUNV-infected animals indicated in Fig 1B (i.e. BUNV s.c. #4 and BUNV i.p. #3) and formalin-fixed before being processed for hematoxylin/eosin staining as well as immunohistochemistry. No lesions were observed for the liver or splenic white pulp; however, consistent with the gross pathological finding of mild splenomegaly in BUNV s.c. #4, the red pulp in this animal exhibited acute neutrophilic splenitis with evidence of congestion (Fig 2A, panel i).

In contrast to the limited remarkable findings in the visceral organs, examination of different regions of the brains collected from diseased BUNV-infected animals exhibited a variety of pathological features. The hypothalamus (Fig 2A, panel ii) showed evidence of acute encephalitis as well as vascular damage (including thrombus formation, vascular necrosis and perivascular hemorrhage). Perivascular cuffing was noted in both the cranial colliculus (Fig 2A, panel iii) and thalamus (Fig 2A, panel iv). The cranial colliculus also showed clear evidence of leptomeningitis. Neuronal and glial cell death were observed in sections from the thalamus, cerebellum and hippocampus (Fig 2A, panels iv, v and vi). Neutrophil infiltration and degeneration were observed to a greater or lesser extent in all brain sections examined, consistent with these diverse indications of inflammation and cell death. Further, immunohistochemical analysis for viral protein using an anti-BUNV N antibody showed robust antigen accumulation in both the cell body and processes of neurons, as shown for sections of thalamus and the cerebellum, where all layers (i.e. molecular, Purkinje and granular) are clearly affected (Fig 2B).

## Discussion

Despite representing an important aspect of the clinical disease associated with various orthobunyavirus infections, including those of the Bunyamwera serogroup, in most cases relatively little is known about the mechanisms underlying neuroinvasion from the periphery into the central nervous system during infection or the development of neuropathology (reviewed in [34]). While small animal models that accurately recapitulate aspects of human disease, which in many cases can also include the symptomatic infection of otherwise healthy immunocompetent adults, would provide a valuable tool for addressing these and other important open questions in bunyavirus research, few such models currently exist for these viruses. Further, those that are currently available are mostly based on direct intracranial inoculation of immunodeficient (e.g. IFN knock-out) or immunologically immature (e.g. newborn, suckling or weanling) animals [17–19]. While these models, nevertheless, can have significant value for the study of neuropathology, they are unable to model neuroinvasion from peripheral infection sites, which represents a critical aspect of the pathophysiology of neurological disease. However, recent work has clearly demonstrated that immunocompetent disease models based on peripheral infection are possible in some cases, such as for Cache Valley fever virus [35] and the recently discovered Ebinur Lake virus [36]. Nonetheless, for Bunyamwera virus, which is the prototype of this group, and the member for which the most molecular tools currently exist, no such models have been reported. Appropriate models for the related Batai virus, and the HF-causing BUNV/BATV reassortant NRIV, are also lacking, and this hampers studies related to how this reassortment event may have contributed to NRIV’s heightened pathogenicity.

In an effort to develop animal models that could support research on these viruses, we examined the potential of BUNV, as well as BATV and NRIV, to cause disease in Syrian golden hamsters following peripheral inoculation. While infection with either BATV or NRIV resulted in, at most, a modest delay in weight gain, we found that BUNV infection resulted in severe weight loss and the development of clear neurological signs that necessitated euthanasia of the affected animals (Fig 1). While the frequency with which severe disease was observed was higher in the s.c. group (67%) than the i.p. group (33%), the time to death and the clinical observations were similar regardless of the route of infection (Fig 1). Consistent with the observed neurological signs of disease, analysis of brain sections showed evidence of encephalitis and meningitis, vascular damage leading to hemorrhage, as well as neuronal cell death (Fig 2). Further, the wide-spread and abundant presence of virus antigen in the brain, including neurons, suggests that the neuropathological effects observed following BUNV infection in hamsters are likely due, at least in part, to direct infection-induced damage, although an additional immunopathological component can certainly not be excluded.

While it is unfortunate in some regards, and particularly from the perspective of reducing animal group sizes, that uniform lethality could not achieved even at relatively high challenge doses by either route tested, clinical disease is still sufficiently frequent following s.c. inoculation (i.e. 67% of infected animals), and the clinical presentation among diseased animals sufficiently consistent, to make this model a useful research tool. However, such studies will certainly have to take into account these mixed outcomes when selecting animal group sizes to ensure that statistical significance with respect to the hypothesis under study can be achieved.

Importantly, the animals used in this study were not infected until 5–7 weeks of age, which is the point where they are reaching the onset of sexual maturity and also approaching their ideal adult weight [37,38], and as such, they can be expected to be fully immunocompetent—an observation that is also supported by their relative resistance to infection with the other viruses used in this study, i.e. BATV and NRIV. Where a more uniform outcome is desirable, one viable option might be to use younger animals, i.e. infection of weanling (3-4-week-old) hamsters–as has been done for several other models of orthobunyavirus infection [39–41]. However, while such an approach might be appropriate for certain applications, it also introduces significant limitations by potentially circumventing consideration of the role of the immune system, whether in controlling virus infection or contributing to immunopathology [34]. As such, for studies of pathogenesis in immunocompetent individuals (which are a significant if often underappreciated target group for infection with Bunyamwera serogroup viruses) where the immune system often plays a key role, and might particularly influence neuroinvasion and/or the development of neurological disease, we anticipate that the maturity of the animals used in this study will represent a significant advantage. For the same reasons, they may also represent a more authentic, and therefore predictive, context in which to test antiviral therapies, especially those that target aspects of the host immune response. A further strength of this study is the use of peripheral routes of infection, which allow the study of neuroinvasion. Indeed, a contribution of infection route to outcome can be seen in comparing the frequency of severe disease between i.p. and s.c. infected animals in our study. In this context it is intriguing that severe disease outcomes were more frequently observed with the s.c. route, which while less invasive also more closely approximates the natural route of infection via insect bite. This suggests that the use of intradermal inoculation, which gives the closest experimental approximation to an insect bite (without the use of live insect vectors), might allow further refinement of the model, although it has to be noted that i.d. inoculation is technically challenging to achieve with consistency, especially in small animal models and under high containment conditions, due to the thinness of the dermal layer.

The observations from this work support previous anecdotal reports that lethal infection of hamsters using Batai virus (strain Calovo) occurs only following intracranial but not peripheral inoculation [20, 22]. Further, it suggests that the lack of any reports on infection of hamsters with NRIV, may also be due to the lack of success of such approaches in generating a model of severe disease. Thus, while it remains possible that lethal infections with these viruses could be achieved with other strains or using routes not tested in this study (or by others), our data suggest that hamsters are not suitable disease models for these pathogens. Nonetheless, we still observed statistically significant reductions in weight gain among BATV (s.c.) and NRIV (s.c. and i.p.) infected hamsters, indicating that they do become infected. As such, infection of hamsters with these viruses may provide a suitable system in which to study pathophysiological processes associated with mild disease outcomes and effective viral control, for instance as a counterpoint to the severe disease seen with BUNV infection. However, this potential needs to be explored more thoroughly in future studies focused on serial sampling for viremia and tissue distribution.

Given the close genetic relationship between NRIV and BUNV, with BUNV being the S and L segment donor for the NRIV reassortant [12,13], it is then rather remarkable that these viruses produce such different clinical outcomes in infected hamsters. One possible interpretation is that the M-segment, provided by BATV, plays a decisive role in this phenotype. However, it must also be considered that the BUNV stain used in this study was the prototype 6547–8 strain. While focusing on this strain for model development makes sense from a technical and experimental standpoint, given that essentially all research on BUNV and all the molecular resources currently available are also based on this strain, its extensive passage history also has to be taken into consideration. Specifically, the isolate available from the WRCEVA has undergone 47 documented passages in sucking mice [42], which might also explain its unique ability among these viruses to cause lethal disease, including neuropathology, in hamsters. However, in order to clarify this issue much more extensive studies will be required, on the one hand using genetically diverse and much less extensively passaged, BUNV isolates to establish the role of a possible pre-adaptation to infection/disease in rodent species, as well as the testing of other BUNV/BATV reassortants with various genome constellations to determine if there is a decisive link between the M-segment encoded BUNV G and a lethal phenotype in hamsters. The BUNV hamster model presented here now provides an appropriate basis for conducting just such studies.

Importantly, regardless of the mechanistic basis underlying the ability of BUNV (strain 6547–8) to infect hamsters, it clearly produces lethal disease with neurological clinical signs. Here, the more severe symptoms observed included delayed righting, tremors of the head or front paws, waltzing and face pressing, and possibly seizure. These symptoms are broadly consistent with those seen during infection of newborn mice with BUNV, which has been reported to cause tremors, disorientation and hind limb paralysis [18]. Also, other hamster models of orthobunyavirus infection that have been established based on the use of younger hamsters (i.e. 3–4 weeks) show a similar range of neurological disease signs. For instance, infection with Melao virus (strain BE AR633512) resulted in the absence of motor coordination, shivering and hind limb paralysis [39], while infection with Oropouche virus (OROV) resulted in neurological symptoms that included walking difficulty, frequent stumbling, and occasional hind limb paralysis [41]. Interestingly, this study also reported that OROV-infected animals without paralysis engaged in prolonged episodes of waltzing [41], similar to what we observed with BUNV infection, and suggesting that this symptom is specific to the neurological damage caused by the infection. Also similar to what we report here for BUNV, peripheral infection of hamsters with either Melao or OROV resulted in infection in the brain, where both virus antigen and pathological changes, including the development of meningitis or meningoencephalitis, were observed [39,41]. Even the non-uniform disease outcome we observed following BUNV infection appears to fit with reports for OROV-infected hamsters, where only 55% of the infected animals developed disease [41]. Nonetheless, additional studies remain needed to more fully characterize BUNV infection in hamsters, including detailed studies of the tissue distribution, as well as the kinetics of viremia and antibody development, associated with infection in animals that either survive or develop lethal neurological disease.

The value for research of high-quality immunocompetent animal models that can recapitulate neuroinvasion from the periphery is well illustrated by recent work on members of the California serogroup (CSG) (reviewed in [34]). Here the epidemiological link between these viruses and neurological disease is clear, and the availability of appropriate models is now allowing the pathophysiological mechanisms associated with neuroinvasion and neuropathology to be increasingly understood [34]. Based on its ability to recapitulate symptoms of severe neurological disease in the majority of infected animals after s.c. inoculation, we anticipate that this hamster model for BUNV infection will now be useful in expanding this type of research to other orthobunyaviruses by facilitating experiments that currently cannot be adequately addressed using existing models. Specifically, we can envision its use in studies directly examining the mechanisms underlying neuroinvasion and neuropathogenesis for Bunyamwera serogroup viruses, as well as in studies of pathogenic determinants for BUNV, particularly in conjunction with existing recombinant BUNVs lacking factors important for innate immune regulation (e.g. NSs) [18,43,44] in order to more clearly define their role in infection in an immunologically competent animal context.

## Supporting information

S1 Fig Survival and weight loss in Batai virus (BATV)-infected hamsters. Groups of 6 Syrian golden hamsters were inoculated by either the intraperitoneal (i.p.) or subcutaneous (s.c.) route with 10^6^ plaque-forming units of BATV. **(A) Survival in infected hamsters.** Kaplan-Meier survival curves show the survival of the animals in each group over time. **(B) Weight loss in infected hamsters.** Change in weight over time is shown for both i.p. infected (left panels) and s.c. infected (right panels) animals as both the group mean ± standard deviation (upper panels), and as individual animal weights (lower panels).
(PNG)

S2 Fig Survival and weight loss in Ngari virus (NRIV)-infected hamsters. Groups of 6 Syrian golden hamsters were inoculated by either the intraperitoneal (i.p.) or subcutaneous (s.c.) route with 10^6^ plaque-forming units of NRIV. **(A) Survival in infected hamsters.** Kaplan-Meier survival curves show the survival of the animals in each group over time. **(B) Weight loss in infected hamsters.** Change in weight over time is shown for both i.p. infected (left panels) and s.c. infected (right panels) animals as both the group mean ± standard deviation (upper panels), and as individual animal weights (lower panels).
(PNG)
